# TRAX-CHEMxt: Towards the Homogeneous Chemical Stage of Radiation Damage

**DOI:** 10.3390/ijms24119398

**Published:** 2023-05-28

**Authors:** Gianmarco Camazzola, Daria Boscolo, Emanuele Scifoni, Alexander Dorn, Marco Durante, Michael Krämer, Valentino Abram, Martina C. Fuss

**Affiliations:** 1 Biophysics Department, GSI Helmholtz Centre for Heavy Ion Research GmbH, 64291 Darmstadt, Germany; g.camazzola@gsi.de (G.C.); d.boscolo@gsi.de (D.B.); m.durante@gsi.de (M.D.); martina-christina.fuss@medaustron.at (M.C.F.); 2 Quantum Dynamics and Control Division, Max Planck Institute for Nuclear Physics, 69117 Heidelberg, Germany; dornalex@mpi-hd.mpg.de; 3 Department of Physics and Astronomy, Heidelberg University, 69120 Heidelberg, Germany; 4 Trento Institute for Fundamental Physics and Applications (TIFPA), National Institute for Nuclear Physics (INFN), 38123 Povo, Italy; emanuele.scifoni@tifpa.infn.it; 5 Institute for Condensed Matter Physics, Technical University of Darmstadt, 64289 Darmstadt, Germany; 6 Department of Mathematics, University of Trento, 38123 Povo, Italy; valentino.abram@unitn.it

**Keywords:** chemical track structure, radical/molecule yields, homogeneous biochemical stage, reaction–diffusion equations

## Abstract

The indirect effect of radiation plays an important role in radio-induced biological damages. Monte Carlo codes have been widely used in recent years to study the chemical evolution of particle tracks. However, due to the large computational efforts required, their applicability is typically limited to simulations in pure water targets and to temporal scales up to the µs. In this work, a new extension of TRAX-CHEM is presented, namely TRAX-CHEMxt, able to predict the chemical yields at longer times, with the capability of exploring the homogeneous biochemical stage. Based on the species coordinates produced around one track, the set of reaction–diffusion equations is solved numerically with a computationally light approach based on concentration distributions. In the overlapping time scale (500 ns–1 µs), a very good agreement to standard TRAX-CHEM is found, with deviations below 6% for different beam qualities and oxygenations. Moreover, an improvement in the computational speed by more than three orders of magnitude is achieved. The results of this work are also compared with those from another Monte Carlo-based algorithm and a fully homogeneous code (Kinetiscope). TRAX-CHEMxt will allow for studying the variation in chemical endpoints at longer timescales with the introduction, as the next step, of biomolecules, for more realistic assessments of biological response under different radiation and environmental conditions.

## 1. Introduction

The physical interactions induced by different ionising radiation types considered relevant for therapy (i.e., photons, electrons, protons, carbon ions [1] and, since last year, helium ions [2]) elicit localised damage in biological targets, which may ultimately lead to cell death. Generally, there are two paths to induce damage, either through direct action against the target molecules, or indirectly via the intermediate production of reactive species originated from the radiolysis of water, constituting about 80% by the weight of living cells [3,4,5,6], or other (organic) molecules in the medium. The ratio between the effect of indirect and total damage strongly depends on the radiation type under consideration [7]. From a 150 MeV proton beam, the indirect action to plasmid DNA is higher than 90% [8], while from a 150 MeV/u helium irradiation, the indirect contribution to cell killing under hypoxic conditions approaches ∼50% [9]. A similar result was also found for a 10 MeV/u carbon beam [10]. In other terms, as far as the indirect action is concerned, the quantity and localisation of clustered radicals and molecules produced represent a major difference between the effects of low and high LET [11].

Given the relevant contribution of the indirect action, it is important to have a deeper understanding of its features, also for heavy ions. Along the path to the final deposition of the desired dose into the tumour, the energy decreases, with a corresponding rise of LET. Therefore, due as well to energy straggling and nuclear fragmentation, a broad distribution of LET values, rather than a single one, should be considered [12]. A consequent aspect is the relevance of comparing the effects of various radiation qualities. By studying how the chemical yields change with LET, a better understanding of the major channels responsible for the indirect damage related to that specific beam quality can be obtained. This will contribute to predicting under which conditions normal tissues are subject to severe toxicities, and to improve treatments via new modalities such as ultra-high dose rate (FLASH) irradiations. The FLASH effect, whose mechanisms are still not fully understood [13,14], is an intriguing biological phenomenon for its normal tissue-sparing feature and the simultaneously unchanged efficacy in tumour control, for both low [15,16] and high [17,18] LET radiations. Since several hypothesised mechanisms imply a relevant role of the chemical stages [13], to explore in detail the onset of the aforementioned effect, as well as to assess its possible variation as a function of different radiation qualities [19], a detailed description of the reaction kinetics featuring the produced radicals could be necessary.

**Figure 1:**
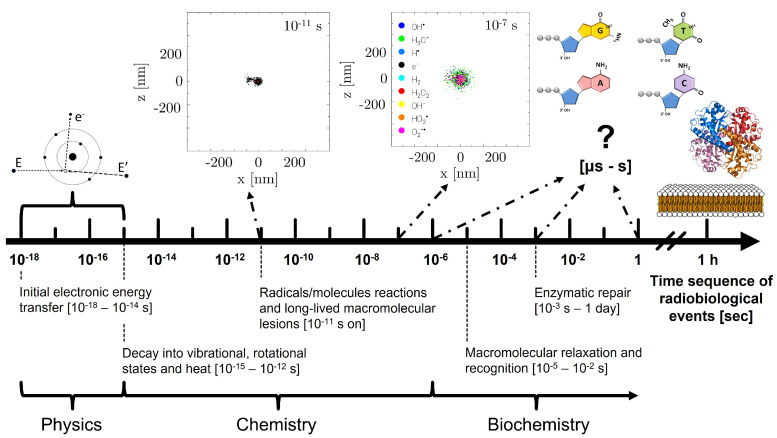
Illustration of the time scales of radiobiological events. In the upper part, examples of chemical track evolution simulations already available in TRAX-CHEM are shown. At the same time, some ingredients that have to be considered to investigate the biochemical stage (DNA nucleotides, enzymes such as superoxide dismutase and lipids) are also depicted.

An analysis of the radiation damage evolution, on a track structure level, may thus provide fundamental insights into its various stages, each characterised by different physical and chemical events. These are schematically summarised in Figure 1. Ionising radiation transfers energy to the water target rapidly, in the order of attoseconds to femtoseconds (physical stage). This induces ionisation and excitation events, which lead to molecular dissociations and the production of sub-excitation electrons, which thermalise with the surrounding environment reaching thermodynamic equilibrium. At approximately 1 ps, the radiolytic products start to behave as chemical reactants, diffusing and interacting between themselves. During this chemical stage, the track structure dynamic rules the chemical evolution of the radicals and molecules, causing a heterogeneous distribution of the species. At later times (∼1 µs), these have diffused away from the track core, reaching a state wherein their concentrations change much more slowly. It is at this point (biochemical stage) that reactions with additional molecules in the biological environment prevail.

Different LET values strongly affect the spatial distribution and heterogeneity of the chemical species produced around the track centre [20,21]. It is, then, appealing to study their behaviours from a computational/theoretical perspective as well. This will allow for an understanding of when standard models based on homogeneous chemistry may be applied, and in which circumstances these become limited or insufficient. Such an analysis is especially relevant for high LET particles, wherein the denser track generated by the primary physical events results in a higher probability of reactions between the species and, consequently, a slower transition to the “classical” homogeneous reaction regime. From the modelling and simulation points of view, different state-of-the-art algorithms for the physical and chemical stages (fs–µs), based on Monte Carlo approaches, already exist: IONLYS-IRT [3], PARTRAC [6], TRAX-CHEM [21], Geant4-DNA [22], TOPAS-nBio [23], DBREAK [24], RITRACKS [25,26], NASIC [27,28] and gMicroMC [29,30], the latter two also being implemented for running on GPUs (for an extensive review, cf. [31]). However, as far as the biochemical stage (µs–s) is concerned, dedicated models have been only recently developed, most of them focusing on implementing a few specific reaction partners [5,32,33]. Simulating the chemical effects induced by radiation in a biological target is, in fact, complicated. The introduction of biomolecular solutes themselves is not trivial and requires information such as biomolecule concentrations, diffusion coefficients and reaction rates that are not always available. Moreover, regardless of the algorithm used to simulate the chemical stage, be it a step-by-step or an independent reaction time (IRT) approach, the introduction of additional species, together with the need to extend the time domain by several orders of magnitude (µs–s), may require to tackle the resolution of the reaction–diffusion equations in a different way for reasons of computational efficiency. In this regard, particular attention must be given to the time point at which the information is handed over from the initial algorithm, designed for heterogeneous modelling, to the new one adapted to a situation that becomes more and more uniform.

The scope of this work is to present TRAX-CHEMxt, the newly developed extension of TRAX-CHEM [34], a chemical integration of the Monte Carlo track structure code TRAX [35,36]. TRAX-CHEM was recently updated to address medium oxygenation [21], which made it possible to gain some mechanistic insights into the role of oxygen in the FLASH effect [37,38]. Nevertheless, a more realistic analysis was limited by the aforementioned restricted timescale accessible. TRAX-CHEMxt can drive the simulation to longer times with improved computational performance while preserving its accuracy. After showing the results that TRAX-CHEMxt can produce in Section 2.1, Section 2.2 and Section 2.3, the validation with the data from standard TRAX-CHEM is presented in Section 2.4.1. Chemical yields obtained with TRAX-CHEMxt are also compared with the outcomes from [20,39], and with the predictions from the fully homogeneous software Kinetiscope (Version 1.1.1136) [40], in Section 2.4.2 and Section 2.4.3, respectively. A general overview of the major results can be found in Section 3, whereas in Section 4 the technical details of the newly developed code are described. Some final discussions and future applications of TRAX-CHEMxt are given in Section 5.

## 2. Results

Thanks to the discrete, step-by-step algorithm applied to compute the pre-chemical and chemical stages of water radiolysis in the full Monte Carlo code (TRAX-CHEM), it is possible to retrieve the position of each individual species produced around the track centre. In order to obtain a simplified, continuous yet not fully homogeneous description of the different chemical yields, the aforementioned information is converted into concentration distributions, one for each radical and molecule. This can be achieved by grouping the number of species in histograms with specific, user-defined radial bin widths, converting those numbers into concentration values. The radial distribution of concentrations results in a chemical extension of the concept of the amorphous track model for the physical radial dose. A new approach to solve the reaction–diffusion equations has been implemented starting from these distributions, which represent the outcome of a “typical” single track, but with on top the possibility to account for neighbouring track effects through appropriate boundary conditions. A mathematical description of the novel method allowing these shortcuts in TRAX-CHEMxt can be found in Section 4.1.

### 2.1. Transition Time and Chemical Track Evolution at the ms Scale

**Figure 2:**
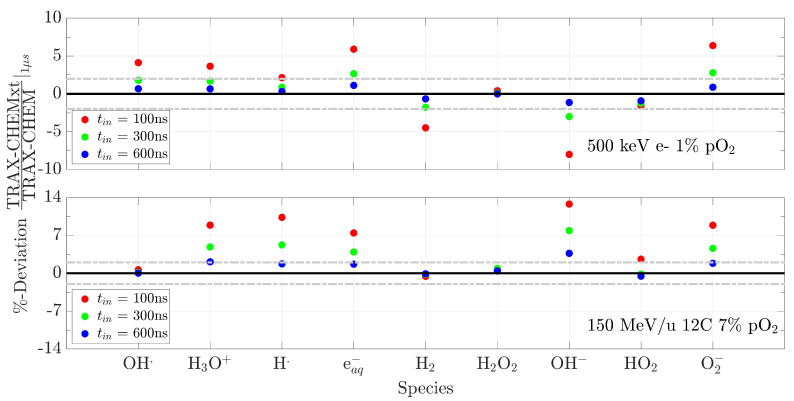
Deviations at 1 µs as a function of the time chosen to pass the species localisation information from TRAX-CHEM to TRAX-CHEMxt. The deviations are determined by the difference between the total number of every radical and molecule predicted by TRAX-CHEMxt and the respective quantity produced by TRAX-CHEM, divided by the latter. Tests on two radiation qualities are shown, namely 500 keV electron beam (**top**) and 150 MeV/u carbon beam (**bottom**), in a water target under pO${}_{2}$ of, respectively, 1% and 7%. Grey dash–dotted lines are introduced to mark deviations of ±2%.

The most relevant parameter that needs to be fixed is the transition time, i.e., the time the information has to be handed over from the full Monte Carlo code to TRAX-CHEMxt, $t_{in}$. Examples of the effects of various $t_{in}$ on the number of species produced at 1 µs, for two radiation qualities and water oxygenation conditions, are presented in Figure 2. Too-short switching times alter the predictions, especially for high LET particles with deviations approaching 15% with respect to the TRAX-CHEM results. In this case, the radicals and molecules are still too close to the track centre and to each other, with a still rather strong dependence on the track structure dynamics. Therefore, values around 500 ns should be preferred to forward the information to the new description with concentration distributions, owing to the small deviations registered (almost all within 2%). From another perspective, this transitional time domain represents a “quasi-continuous” regime, where species have diffused away from the track core, and where a reasonable homogeneity along the track direction, and locally within each radial bin, has been reached.

**Figure 3:**
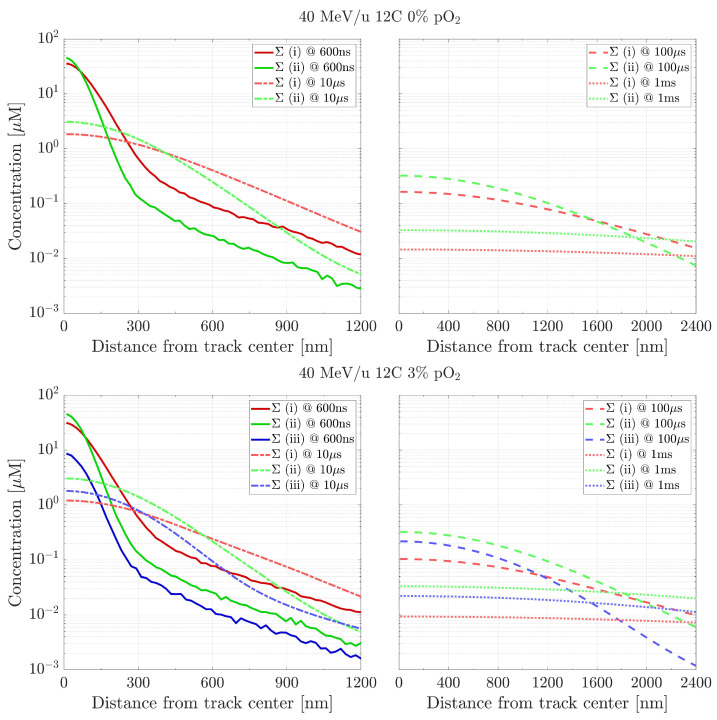
Distributions’ sums, at different time points, of the three species categories produced by a 40 MeV/u carbon beam in 0% pO${}_{2}$ (**top**) and 3% pO${}_{2}$ (**bottom**) water targets. Category (i): $OH^{•}$, $H_{3}O^{+}$, $H^{•}$ and $e_{\mathrm{aq}}^{-}$; category (ii): $H_{2}$, $H_{2}O_{2}$ and $OH^{-}$; category (iii): $HO_{2}^{•}$, $O_{2}^{•-}$ and $HO_{2}^{-}$. Initial data taken at $t_{in}$ = 600 ns.

Once the proper transitional time is set, longer time scales can be probed. For simplicity, the species are here grouped into three different categories, subdivided as follows. The first one (i) represents the typical direct products of water radiolysis ($OH^{•}$, $H_{3}O^{+}$, $H^{•}$ and $e_{\mathrm{aq}}^{-}$). The second (ii) refers to the intra-track recombination molecules ($H_{2}$, $H_{2}O_{2}$ and $OH^{-}$), whereas the last category (iii) accounts for the products of the reactions with oxygen dissolved in the target medium ($HO_{2}^{•}$, $O_{2}^{•-}$ and $HO_{2}^{-}$). Figure 3 shows the radial distributions of these quantities, produced by a 40 MeV/u carbon beam up to 1 ms and under two different pO_2_ values, 0% and 3%. The fluctuations at 600 ns are due to the intrinsic statistical fluttering of the species when imported from TRAX-CHEM in a histogram format. In both oxygenation conditions, the concentrations approach a flat value only at later stages, approximately around 1 ms, whereas at 100 µs they still exhibit a radial dependence. As time passes, the concentrations of the first category radicals (i) decrease, in favour of an increase in the products from intra-track (ii) and oxygen (iii) reactions.

**Figure 4:**
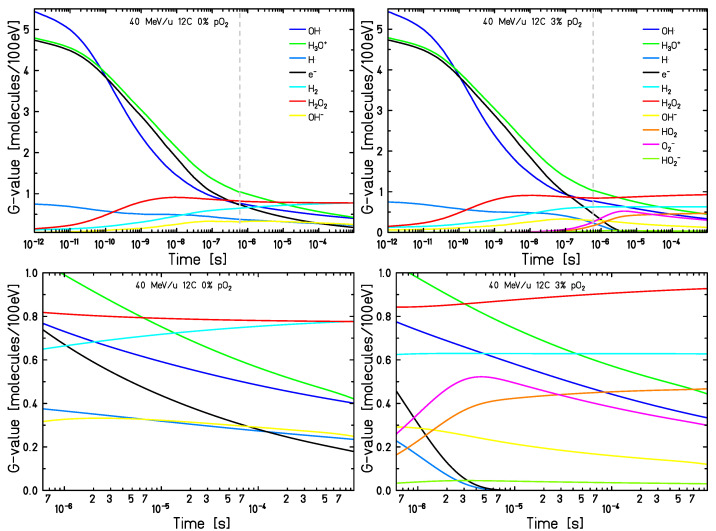
G-values up to 1 ms of the species produced by a 40 MeV/u carbon beam in 0% pO${}_{2}$ (**left**) and 3% pO${}_{2}$ (**right**) water targets. In the bottom row, insets between 600 ns and 1 ms of the curves presented in the top row are reported. Initial data taken at $t_{in}$ = 600 ns (grey dashed lines).

The G-values of the aforementioned categories, representing the number of radicals or molecules produced in the target per 100 eV of energy deposited, are displayed in Figure 4, for the same irradiation and environmental conditions. The number of intra-track products increases as time passes. Moreover, in oxygenated conditions, $H^{•}$ and $e_{\mathrm{aq}}^{-}$ are fully consumed at earlier times, as already observed in [20,21]. While at 0% pO_2_ hydrogen peroxide seems to have reached a steady state, at 3% its G-value rises with time. A slight increase could be caused by the rearrangement of the information from TRAX-CHEMxt itself (cf. Section 3). However, a similar trend was observed with the standard version of the TRAX-CHEM code when running up to 10 µs, suggesting a direct involvement of the reaction network (Table S1 in the Supplementary Materials). The main channel responsible for $H_{2}O_{2}$ production is $OH^{•}+OH^{•}\to H_{2}O_{2}$. In anoxic conditions, this competes with $OH^{•}+e_{\mathrm{aq}}^{-}\to OH^{-}$. When oxygen is present in the environment, the majority of hydrated electrons react with $O_{2}$ to produce $HO_{2}^{•}$, leaving more hydroxyl radicals the chance to recombine and generate $H_{2}O_{2}$. The other reactions involved in the production of hydrogen peroxide, namely $H^{•}+HO_{2}^{•}$ and $H_{3}O^{+}+HO_{2}^{-}$, have been shown to have a negligible effect. Overall, the species that were thought to have reached a “saturation” level at 1 µs, are instead still registering some fluctuations around this time scale.

### 2.2. LET Impact

**Figure 5:**
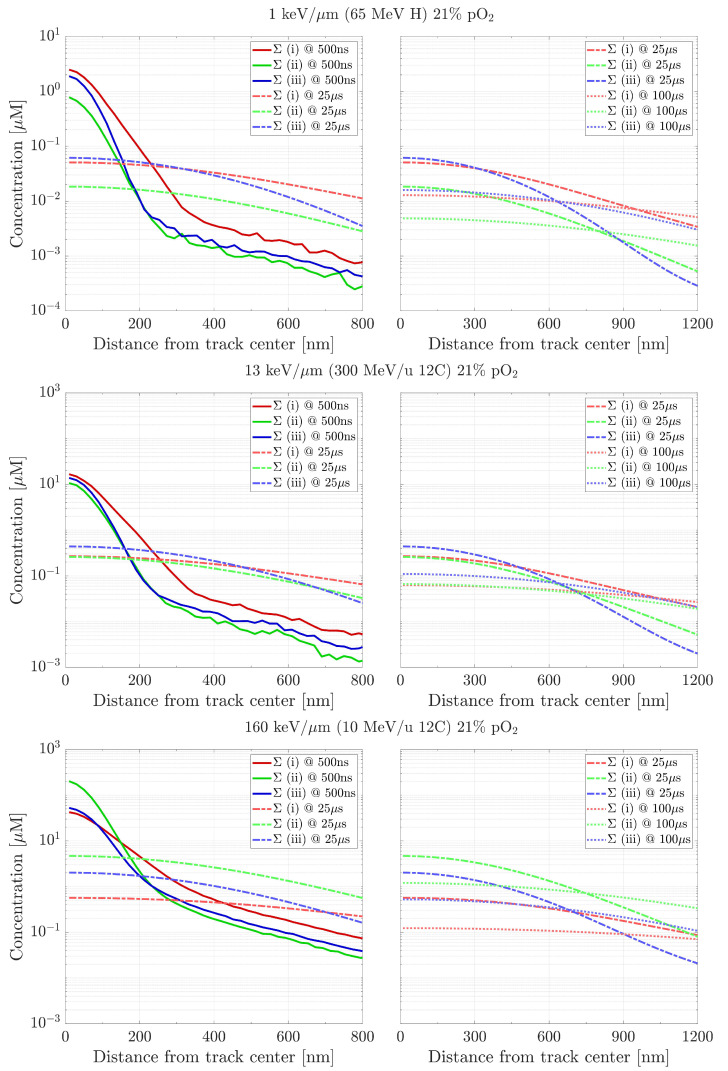
Distributions’ sums, at 500 ns, 25 µs and 100 µs, of the three species categories for increasing LET values, respectively, ∼1 keV/µm, ∼13 keV/µm and ∼160 keV/µm, under pO${}_{2}$ = 21%. Category (i): $OH^{•}$, $H_{3}O^{+}$, $H^{•}$ and $e_{\mathrm{aq}}^{-}$; category (ii): $H_{2}$, $H_{2}O_{2}$ and $OH^{-}$; category (iii): $HO_{2}^{•}$, $O_{2}^{•-}$ and $HO_{2}^{-}$. Initial data taken at $t_{in}$ = 500 ns.

Figure 5 shows the LET impact on the radial distributions of the three different categories in a water target at 21% oxygenation. The chemical species’ concentrations increase with LET. In particular, for high LET radiations, a larger production of intra-track molecules (ii) is observed at all time points, suggesting a more frequent recombination of radicals during the early stages of the chemical evolution. For the low LET radiation, instead, a predominance of primary radicals (i) and products from reactions with oxygen (iii) are registered. At 100 µs, the average molecules’ concentration (ii) seems to scale linearly with LET, passing from approximately 4 nM for the lowest LET investigated (∼1 keV/µm), to ∼700 nM for the highest one (∼160 keV/µm). At the same time, the distance from the track centre at which these species (ii) surpass the radicals (i) decreases as the LET increases, and while similar trends during the heterogeneous chemical stage were already pointed out in previous works [19,21], the present finding is showing the significant persistence of the LET impact at longer timescales.

**Figure 6:**
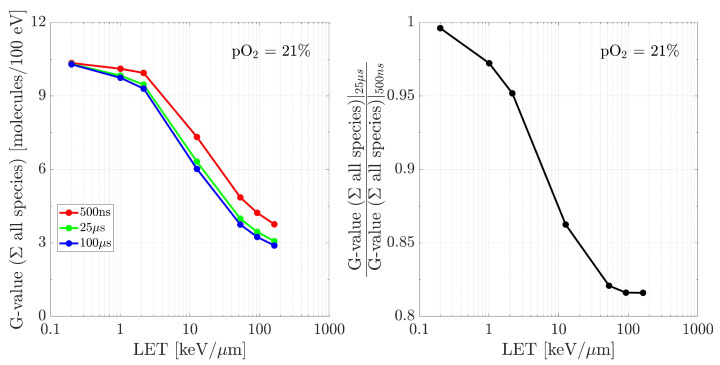
(**left**) G-values of all the species produced by different LET radiations, recorded at 500 ns, 25 µs and 100 µs. (**right**) Ratios between the G-values at 25 µs and 500 ns. The environment was kept at pO${}_{2}$ = 21%. Initial data taken at $t_{in}$ = 500 ns.

The cumulative G-values computed at different time points as a function of LET are shown in Figure 6. Although the absolute number of species grows with LET due to the larger energy deposited, their respective G-values decrease. Indeed, the recombination of the chemical species is larger due to track density effects. Moreover, the overall G-value for very low LET radiations (<1 keV/µm) does not change over time, with a ratio between the amounts of 25 µs and 500 ns close to 1. On the contrary, as the LET increases, so do the divergences between the time points, registering a decrease of almost 20% for a very high LET. This suggests that the time at which the reaction network approaches an equilibrium is strongly LET-dependent. In conclusion, it can be inferred that the choice of $t_{in}$ is indeed relevant for these radiations, with 500 ns being at the acceptability border, by noticing how much the G-values drop between 500 ns and 25 µs.

### 2.3. Oxygenation Impact

**Figure 7:**
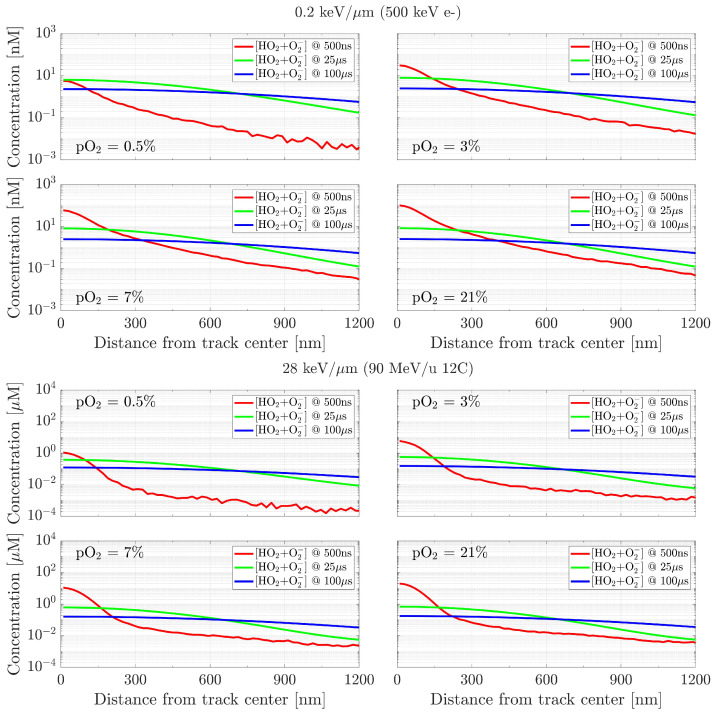
Effects of target oxygenation on the concentration distribution of ($HO_{2}^{•}+O_{2}^{•-}$), recorded at three time points (500 ns, 25 µs, 100 µs), for a low (∼0.2 keV/µm, **top**) and a high (∼28 keV/µm, **bottom**) LET radiation. Initial data taken at $t_{in}$ = 500 ns.

In this section, the impact of different oxygenation levels on the chemical yields at longer times is investigated. In Figure 7, the results are reported for a low (∼0.2 keV/µm) and a high (∼28 keV/µm) LET radiation, for the two major products of reactions with $O_{2}$, namely $HO_{2}^{•}$ and $O_{2}^{•-}$. As the pO_2_ increases, the concentrations’ fluctuations at the initial time (500 ns) decrease, due to the higher number of products present. At 100 µs and under pO_2_ = 21%, the distribution for the low LET radiation tends to become uniform at approximately 1 nM, whereas the one from high LET does so at around 100 nM. For both particle types, even if at 500 ns different concentrations are registered depending on the initial oxygenation, at longer times the distributions tend to stabilise to similar values, independently of pO_2_.

**Figure 8:**
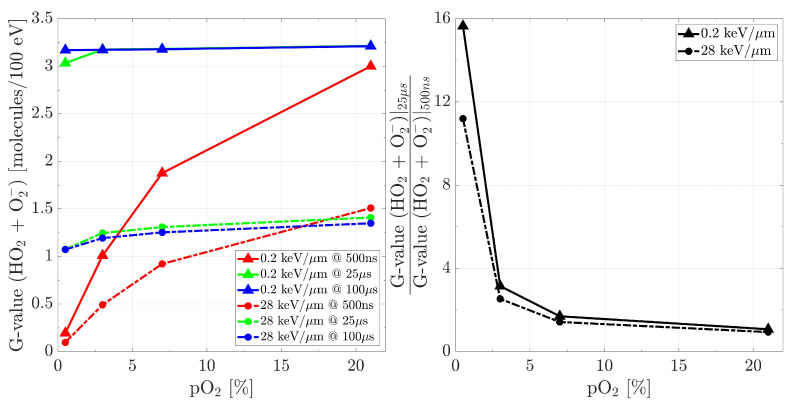
(**left**) G-values for ($HO_{2}^{•}+O_{2}^{•-}$) as a function of pO${}_{2}$, recorded at 500 ns, 25 µs and 100 µs, from radiations with LET of ∼0.2 keV/µm and ∼28 keV/µm. (**right**) Ratios between the G-values at 25 µs and 500 ns. Initial data taken at $t_{in}$ = 500 ns.

G-values for the couple ($HO_{2}^{•}+O_{2}^{•-}$), as a function of environment oxygenation at three time points (500 ns, 25 µs, 100 µs), are reported in Figure 8 for both ionising radiations. Different “saturation” levels are registered, namely 3.2 and 1.4, for an LET of ∼0.2 keV/µm and ∼28 keV/µm, respectively. These values are reached at approximately 25 µs, with the exception of small deviations for very low oxygenation conditions. Furthermore, within the same time gap, the species’ amounts increase differently depending on the beam quality. Under the same (low) oxygenation, at a low LET the G-values grow by almost 16 times when moving from 500 ns to 25 µs, whereas those for the high LET radiation increase by only a factor 11. As the oxygenation grows, these increments diminish, and the difference between the two particle types attenuates as well.

### 2.4. Comparisons with Previous Approaches

#### 2.4.1. Comparison with TRAX-CHEM (Monte Carlo Domain)

**Figure 9:**
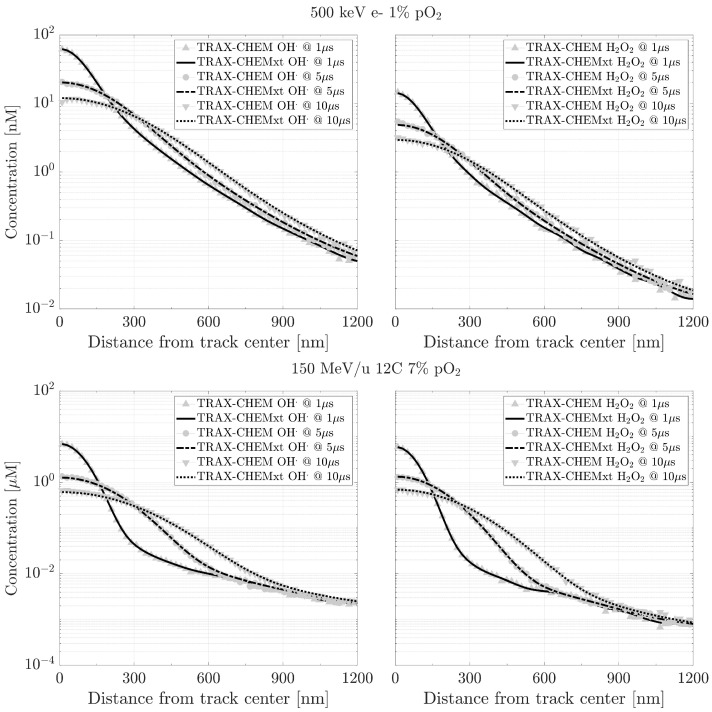
Examples of compared radial distributions for $OH^{•}$ and $H_{2}O_{2}$, produced by a 500 keV electron beam (**top**) and a 150 MeV/u carbon beam (**bottom**), under oxygenation conditions of, respectively, 1% and 7%. Initial data taken at $t_{in}$ = 600 ns.

**Figure 10:**
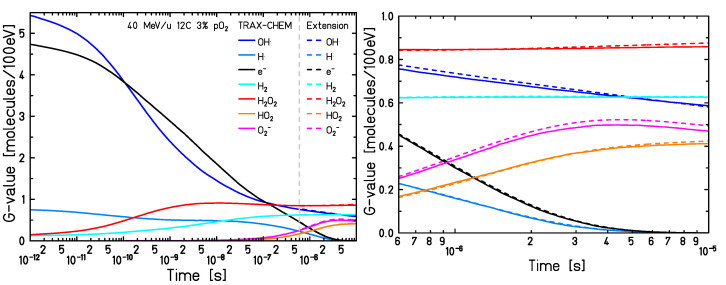
Example of compared G-values for the species produced by a 40 MeV/u carbon beam in a water environment with pO${}_{2}$ = 3%, up to 10 µs. On the right side, an inset to visualise the differences within the overlapping time scale 600 ns–10 µs. Initial data taken at $t_{in}$ = 600 ns (grey dashed line).

The predictions of TRAX-CHEMxt are compared with those from the standard TRAX-CHEM code, in the overlapping time scale (500 ns–10 µs), for different beam qualities and oxygenation conditions. In Figure 9 and Figure 10, examples of radial distributions and G-values are shown. A good agreement is found for all the chemical species studied. This proves that TRAX-CHEMxt is able to carry on the information provided by the Monte Carlo algorithm to longer time scales with a lower computational effort while preserving its accuracy. Additionally, it can be confirmed from another perspective that the choice of the time point for a smooth transition between the two approaches, around 500 ns, is valid.

The validity of TRAX-CHEMxt is also tested with a different approach. In Table 1, the yields at 1 µs of ($H^{•}+e_{\mathrm{aq}}^{-}$) in anoxia and ($HO_{2}^{•}+O_{2}^{•-}$) in 21% pO_2_, from two low LET radiations, 500 keV electrons and 90 MeV protons, are compared. Due to the reactions $e_{\mathrm{aq}}^{-}+O_{2}\to O_{2}^{•-}$ and $H^{•}+O_{2}\to HO_{2}^{•}$, respectively, (xiv) and (xv) in Table S1 of the Supplementary Materials, it is expected that the total number of reactants turns into the respective products when oxygen is present. The resulting yields agree within 1%. For completeness, the respective outcomes calculated for a high LET radiation, 90 MeV/u carbon beam, are also shown. In this case, the discrepancies between the two codes are marginally larger (11%), due to an increased reaction kinetics between the radicals within the track. For a complete overview of the %-deviations found when comparing the two algorithms, refer to Table S2 of the Supplementary Materials. All the values obtained are below 6%, with the highest deviations registered, within the same beam quality, for high oxygenations, and with a further increase from low to high LET radiations. This increasing disagreement is likely due to different track dynamics featuring the various beam qualities, originating from the initial time chosen to switch the information between TRAX-CHEM and TRAX-CHEMxt. Furthermore, additional contributions may come from the possible production of secondary electrons (cf. Section 3).

Compared to the pure Monte Carlo step-by-step approach, with TRAX-CHEMxt a major improvement in the computational effort is registered. A summary of the simulation times (τ) required from both algorithms to obtain the data in Figure 9, together with the relative computational gains (>3 orders of magnitude), is presented in Table 2. The computational gain refers to the physical computing time saved while using TRAX-CHEMxt, in order to reach the same final conditions, due to the spatial concentration distribution simplification. Even if the difference is evident, it must be pointed out that, whereas TRAX-CHEM is a fully Monte Carlo code, TRAX-CHEMxt is based on a numerical approach. The two models are indeed designed to work within two different spatiotemporal regimes.

#### 2.4.2. Comparison with Experimental Data and a Different Monte Carlo Algorithm

The TRAX-CHEMxt outcomes have also been validated with the experimental data presented in [39]. For this purpose, yields of $OH^{•}$, ($HO_{2}^{•}$ + $O_{2}^{•-}$) and $H_{2}O_{2}$ produced by a 500 keV electron beam—since the current version of the TRAX code does not allow for simulations of photon radiation—are computed. Electrons are used due to their similar LET (0.2 keV/µm) with the γ-Co${}^{60}$ source. Moreover, as shown by several authors [26,41], these two radiations result in similar G-values, given the former to be under “track-segment conditions”, i.e., constant LET within the simulated volume. The chemical yields obtained for a pO_2_ of 21% are summarised in Table 3. TRAX-CHEMxt predictions agree well with both the experimental data and another Monte Carlo code exploiting an IRT algorithm [20].

**Figure 11:**
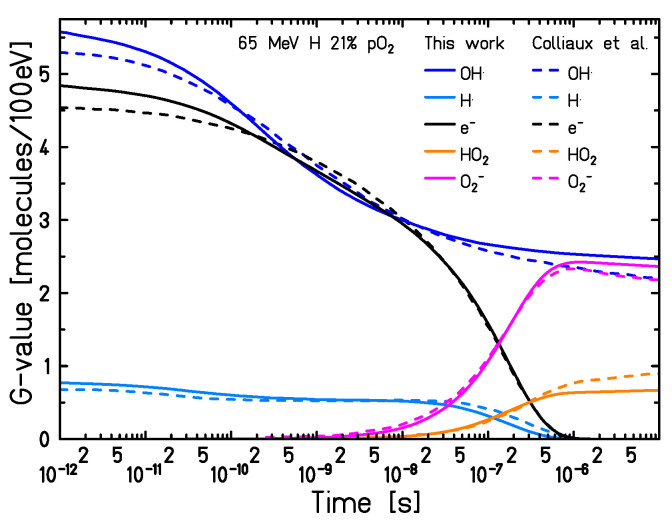
G-values of the main species produced by a 65 MeV proton beam in a water environment with pO${}_{2}$ = 21%. Solid lines correspond to the results from TRAX-CHEM and TRAX-CHEMxt, whereas the dashed ones to those from Colliaux et al. [20]. The extension took over the simulation at 500 ns.

Additionally, G-values have also been compared with those obtained by the Monte Carlo IRT method [20]. Results are shown in Figure 11 for a 65 MeV proton beam in a 21% oxygenated water environment. The curves show some discrepancies, already at 1 ps, mainly due to different physical and pre-chemical models adopted by the two codes, as detailed in [34]. These result in distinct spatial distributions of the species produced around the primary radiation, and thus in diverse developments of the reaction network. In addition, substantial differences are present between the resolution algorithms applied to compute the track evolution during the heterogeneous chemical stage. On one side an independent reaction time, and on the other a combination of a step-by-step and a diffusion–reaction approach with concentration distributions. Overall, the biggest deviations are just on the order of 0.3 molecules/100 eV in the cases of $OH^{•}$ and $HO_{2}^{•}$.

#### 2.4.3. Comparison with Kinetiscope (Pure Homogeneous Domain)

**Figure 12:**
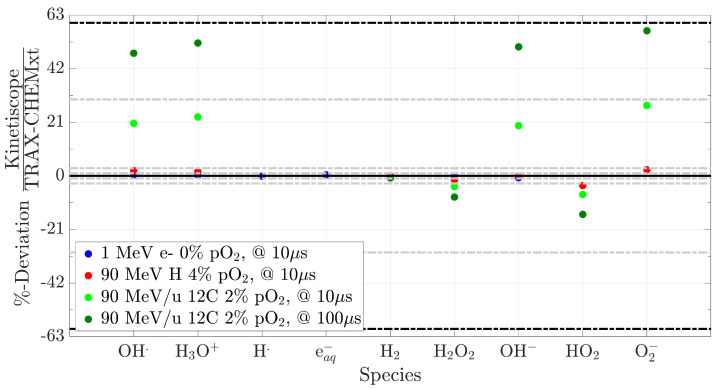
Deviations between Kinetiscope and TRAX-CHEMxt, registered for different beam qualities, oxygenation conditions and final time points. Marked with dash–dotted lines are discrepancies of ±1%, ±3%, ±30% and ±60%. Initial data handed over to both codes at 1 µs.

In this final section, the hypothesis of supposing a fully homogeneous approach from 1 µs onwards to simulate the reaction network, regardless of the initial beam quality, is investigated. To this aim, the predictions of TRAX-CHEMxt are compared with those from Kinetiscope [40], an online software based on a fully homogeneous stochastic model to compute the reactions. The kinematic scheme featuring the evolution of the system is determined by a random selection of probability-weighted reaction channels. Each species is assigned one constant concentration, in order to have a homogeneous composition featuring the reactants in the domain. To perform the comparison, data from TRAX-CHEM at 1 µs are handed over to both software. For Kinetiscope, the uniform concentrations are obtained by dividing the number of radicals and molecules by *A*, the surface around the track, with $A=\frac{1}{\Phi }$ and Φ being the particle fluence (particles/cm^2^). A dose rate $\dot{D}$ of ∼1 Gy/min is supposed, and different beam qualities are tested, with fluences typically applied in conventional treatment plans, namely $10^{7}$ particles/cm${}^{2}$, $10^{9}$ particles/cm^2^ and $10^{10}$ particles/cm${}^{2}$ for carbon ions, protons and electrons, respectively. Starting from these values, fluences at the µs time scale are computed and then converted into the respective surfaces. At final times, 10 µs or 100 µs, the differences between the two algorithms are determined from the number of species predicted. The results of this comparison are shown in Figure 12. The homogeneous approximation at 1 µs proves to be valid for low LET radiations, demonstrating at the same time the consistency and reliability of the present code when appropriate conditions are fulfilled. In these cases, the deviations between the two algorithms at 10 µs do not reach 1% for 1 MeV electrons, whereas a maximum of 4% is registered for a 90 MeV proton beam. However, as both the LET and the final recording time increase, so does the disagreement. For a 90 MeV/u carbon beam, a maximum discrepancy of ∼30% is registered at 10 µs, approaching almost 60% at 100 µs. This is a strong indication that, to reliably simulate a wider range of initial conditions, the species track structure dependence needs to be explicitly accounted for, still at the µs time scale but in a less “localised” fashion. TRAX-CHEMxt does so through the use of radial concentration distributions, while fully homogeneous codes can not manage the description under these conditions. This holds especially for intermediate and high LET radiations, where the (local) steady states for the radicals and molecules are still not fully reached at the end of the heterogeneous chemical stage. In conclusion, for a complete investigation proving that both software provide the same results in a reaction-limited domain, a “dummy” case with uniform concentrations assigned randomly to each chemical species, in a water environment with pO${}_{2}$ = 1%, is depicted in Figure S1 in the Supplementary Materials. Within this homogeneous assumption for the radicals and molecules, all the deviations registered are below 1%.

## 3. Discussion

The aim of this work is to prove the validity of TRAX-CHEMxt, the new extension of the track structure code TRAX-CHEM for several beam qualities, LET values and oxygenation conditions. It demonstrated its capability to carry on the simulations to longer time scales, up to the ms as shown in Figure 3 and Figure 4, therefore with the possibility of exploring the homogeneous biochemical stage. A different development in the G-values for $H_{2}O_{2}$ under anoxic and oxic conditions is found. The competing reactions completed at hundreds of ns reveal their effects at the ms time scale, suggesting that hydrogen peroxide will reach a uniform yield only at later points, around 2 ms, when oxygen is present. Hence, the opposite (decreasing) trend registered for $H_{2}O_{2}$ at ultra-high dose rates [42,43] leads to considering the possible further interplay of other reaction channels featuring the chemical network. A precise description of these dynamics at longer times may be very precious because it could elicit effects of secondary and slower reactions which are not perceived at early stages. Probing scales beyond the µs gave insights also in terms of radicals’ and molecules’ dependence on LET and oxygenation. A possible linear relationship between the average intra-track products concentration (category ii) and LET is registered at 100 µs (Figure 5), suggesting that the time at which the reaction network approaches an equilibrium is LET-dependent as well (Figure 6). On the other hand, under very hypoxic conditions, low LET radiations induce a higher increase in the G-values of the species ($HO_{2}^{•}+O_{2}^{•-}$) compared to high LET ones (Figure 8).

The reliability of TRAX-CHEMxt relies on a computationally efficient algorithm, based on concentration distributions and featured by a computational gain of more than 3 orders of magnitude (Table 2). This has been proven by obtaining an acceptable agreement with experimental data, cf. Table 3, together with values compatible with those from TRAX-CHEM, both in terms of radial concentrations and G-values (Figure 9 and Figure 10). These results suggest that the approximation used for a transitional time at around 500 ns is acceptable, even if for high LET radiations it elicits higher deviations (Table S2 in the Supplementary Materials). This value relates well with that obtained by applying the formula proposed by [44] using an appropriate set of parameters, together with other observations related to the end of the spur expansion [5,26,45]. However, it strongly depends on the radiation simulated, as well as on the chemical species under analysis [41,44]. The higher the number of species produced close to the track core, the higher the recombination rate, and the later the local homogeneity between the different histogram bins reached. The relevance of the choice of an appropriate $t_{in}$ was reported in both [26,41], where it was underlined how the uncertainty associated with determining the value of the transitional point between heterogeneous and homogeneous chemistry, for different LET values, results in differences between the model’s predictions and experimental data. Therefore, in principle, it may be ideal to find an appropriate switching time for each beam quality and initial energy. As a consequence, the method used in TRAX-CHEMxt to convert the information from a discrete approach into radial concentrations has an uncertainty, due to the initial clustering of the species. This represents the intrinsic uncertainty of the new resolution algorithm.

**Figure 13:**
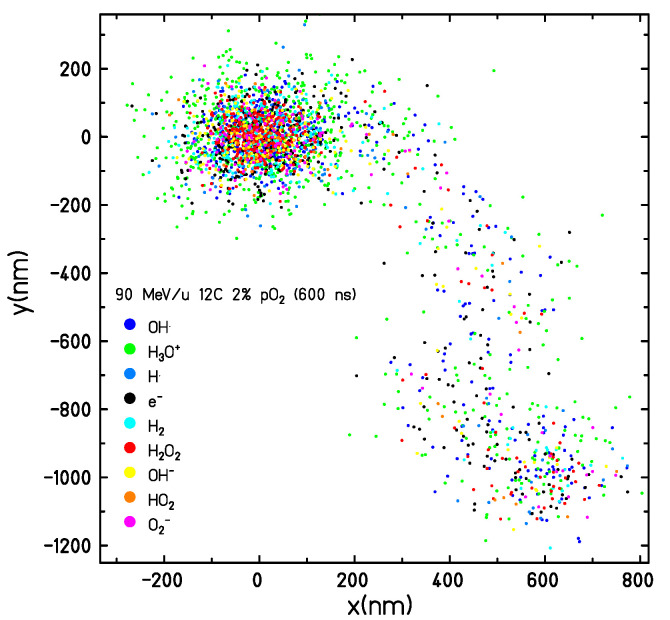
Species produced by a 90 MeV/u carbon ion in a water target with pO${}_{2}$ = 2% (generated by TRAX-CHEM). The screenshot was taken at 600 ns from the initial physical interactions. Clearly visible is the effect of a secondary electron resulting in an energy deposition away from the track center.

A possible additional cause of the deviations registered can be related to the emission of secondary electrons from water molecules. By depositing energy away from the track centre, these events will induce the formation of localised clusters of species, “breaking” the local radial symmetry, as can be seen in Figure 13. Therefore, by grouping the radicals and molecules in different histogram bins instead of treating each singular one separately, they are “smeared out” in the various rings around the track core, slightly reducing their consumption rate. Nevertheless, if these uncertainties are obtained with an approach not completely homogeneous, by extrapolation it is expected that the predictions produced by a completely homogeneous method do not improve (cf. Section 2.4.3). Looking at the specific species ratios in Figure 2, those showing a large disagreement with TRAX-CHEM, especially for the carbon case and independently of the $t_{in}$ chosen, are the ones for $H_{3}O^{+}$ and $OH^{-}$. These molecules are characterised by the highest diffusion coefficients and the highest rate constant ($H_{3}O^{+}+OH^{-}\to H_{2}O+H_{2}O$, reaction (xiii) in Table S1 of Supplementary Materials). Hence, they have not reached a local stabilisation at the transition time. Similar arguments can be moved to explain the discrepancies registered for the other radicals.

TRAX-CHEMxt results reliably match both those from Monte Carlo approaches in the heterogeneous chemical stage and the ones from pure homogeneous codes at low LET. Vice-versa, for intermediate and high LET values, discrepancies higher than 20% with the fully homogeneous approximation appeared (Section 2.4.3). The consideration of a second “intermediate” phase, where the dependence of the species’ positions around the track is still accounted for, is therefore necessary for trusting the simulation outcomes. However, for an optimal analysis of the indirect damage via the exploration of the biochemical stage, some improvements must be made. Additional biomolecules have to be considered. Primary candidates are lipids such as liposomes, a good proxy for cell membranes [46]; amino acids; DNA/plasmids and free nucleotides; metallic ions participating in the Fenton reaction; antioxidants like superoxide dismutase (SOD), catalase (CAT), glutathione (GSH) and ascorbate. Moreover, accounting for possible pH changes which affect both reaction rate constants and acid-base equilibria, could also improve the precision of the simulation.

## 4. Materials and Methods

### 4.1. Code Implementation

**Figure 14:**
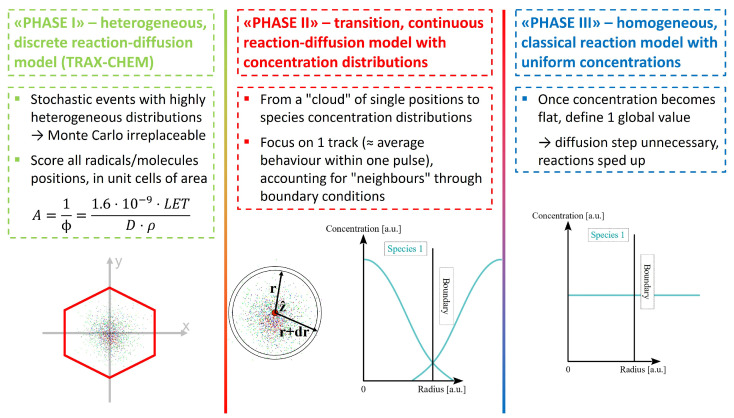
Scheme of the different “phases” featuring the extended simulation. After following every single radical and molecule produced around the track centre, the information is converted into one concentration distribution per species. In the end, once these concentrations reach a uniform value, the classical homogeneous reaction model comes into play.

The development of TRAX-CHEMxt relies on the capabilities of the TRAX-CHEM code [34], which in turn is a chemical extension of the classical physics event-by-event Monte Carlo software TRAX [35,36]. This track structure algorithm simulates the physical and heterogeneous chemical stages of radiation damage with a step-by-step approach. For each ionised/excited state of the water molecules, following specific branching ratios, the radicals are formed at 1 ps. The species then diffuse, with steps sampled from 3D Gaussian distributions with displacements determined by the Einstein–Smoluchowski equation, and at the same time react with each other through the definition of proximity parameters (“reaction radii”). With a recent update [21], it is also possible to include molecular oxygen as an additional reacting partner in the water environment. Differently to IRT methods [25], this mechanism allows for defining, at each simulation time step, the exact position of all the chemical species generated along the primary irradiation, at the cost of a higher computational effort. With TRAX-CHEMxt, the amount of information that has to be carried on during the calculation is reduced. However, differently from the approach exploited in [44], the radicals and molecules are still recorded with respect to the track centre, but in a less localised fashion. To have a clearer picture in mind, three different “phases” for the extended simulation can be discerned, as depicted in Figure 14. The starting “phase I” represents the heterogeneous chemical stage, where the species’ distributions are not homogeneous, not even locally, and the Monte Carlo approach is irreplaceable [5,24,47]. After this stage, all the single positions are scored within an area determined through the dose–LET relation (cf. Equation (4)). “Phase II” is a transitional phase, where a local homogeneity for the various species can be supposed. In this stage, the information is converted from discrete positions to radial concentrations, used to describe each radical and molecule. These distributions are binned as histograms. Reaction and diffusion processes, in a geometry wherein the track structure dependence is still present, are carried on under the assumption that the species’ concentration within each histogram bin is uniform. For a better understanding of how the information is converted before being handed over to TRAX-CHEMxt, refer to Figure S2 in the Supplementary Materials. In principle, one should take into account the radiation quality simulated, as well as the time point at which the concentration distributions are considered. However, a thorough analysis was performed to find a general value for the bin size, applied to all particles simulated (cf. Section 4.3). Effects of possible neighbouring tracks are also considered, via the boundary conditions imposed on the reaction–diffusion problem (seen later in this section). However, it is important to point out that, in this analysis, for both TRAX-CHEM and TRAX-CHEMxt, the chemical effects of the species produced by one single track were studied. Crucial for this phase is the choice of the transitional time at which the Monte Carlo information is converted into the distribution representation. Finally, there is the third phase, “phase III”, wherein the various species’ distributions become flat, and the classical homogeneous reaction model is applied without the need for computing the diffusion.

Due to the novel representation of the chemical species, a new approach is required to numerically solve the partial differential equations featuring the reaction and diffusion events within the system. The problem is divided into a pure diffusion part and a pure reaction part, resolved pairwise and alternatively for each time step. This is due to two main reasons. First, the reaction part is non-linear and highly coupled (e.g., reactions between two chemical species of the same type), and thus it does not allow for a proper treatment in finite difference schemes. Second, in order to conserve the particle concentrations, it is necessary to perform the reactions accurately and at the same time step. Therefore, it is not feasible to linearise non-linear reactions in order to incorporate them into the linear diffusion solution strategy. Concerning the diffusion part, this is described by applying the method proposed in [48]. The initial-boundary value problem associated with Fick’s second law on a cylindrical domain “Dom” of radius $r_{max}$, for the species *A* characterised by a diffusion coefficient *D* is

(1) $$\left\{\begin{array}{c} \partial_{t}\left[A\right]\left(t,\vec{x}\right)-D\Delta \left[A\right]\left(t,\vec{x}\right)=0\\ \left(t=0,\vec{x}\right)=\left[A_{0}\right]\left(\vec{x}\right)\;\mathrm{Initial}\mathrm{condition}\\ \mathrm{Boundary}\mathrm{conditions}\mathrm{at}\mathrm{the}\mathrm{boundary}\mathrm{of}\mathrm{Dom} \end{array}\right.$$

Assuming a cylindrical symmetry of the solution, with [A](t,r) symbolising the concentration distribution as a function of time *t* and of the distance from the track centre *r*, the initial-boundary value problem becomes

(2) $$\left\{\begin{array}{c} \partial_{t}\left[A\right]\left(t,r\right)-D\partial_{r}^{2}\left[A\right]\left(t,r\right)-\frac{D}{r}\partial_{r}\left[A\right]\left(t,r\right)=0\\ \left[A\right]\left(t=0,r\right)=\left[A_{0}\right]\left(r\right)\;\mathrm{Initial}\mathrm{condition}\\ \mathrm{Boundary}\mathrm{conditions}\mathrm{at}r=0\;\mathrm{and}\;r=r_{\max} \end{array}\right.$$

The initial condition, that is, the concentration distributions given at the initial time t=0, is provided by the TRAX-CHEM code. On the other hand, concerning the boundary conditions (B.C.) at the extremes, at *r* = 0 a consistency requirement is imposed. As the solution of Equation (1) is known to be smooth on the interval [ϵ,∞)×Dom for every ϵ>0 [49], a consistency requirement is needed in the cylindrical coordinate formulation under the assumption of angular symmetry, to ensure smoothness at the origin. This requirement is $\partial_{r}{\left[A\right]\left(t,r\right)|}_{r=0}=0$. On the other hand, at *r* = $r_{max}$, a Neumann B.C. is introduced, that is, the value of the derivative of the function at the boundary of the domain is specified. Radicals and molecules are free to diffuse and react; however, due to symmetry considerations in a parallel, uniform radiation field, the number of chemical species leaving the surface delimited by $r_{max}$ is equal to the amount produced by other neighbouring tracks entering into it. Therefore, a net null flux can be supposed at the border, i.e., $\partial_{r}{\left[A\right]\left(t,r\right)|}_{r=r_{max}}=0$. For all the cases studied in this first analysis, $r_{max}$ is set large enough, i.e., 5 µm, to avoid any of these effects at the border since the study of possible intertrack interactions is beyond the scope of this paper. Generally, $r_{max}$ can be computed by the dose–LET relation. The initial expression is

(3) $$D\left[Gy\right]=1.6\cdot 10^{-9}\cdot LET\left[keV/µm\right]\cdot \Phi \left[particles/cm^{2}\right]\cdot \frac{1}{\rho }\left[\frac{cm^{3}}{g}\right]$$

where D is the dose, Φ is the particle fluence and ρ is the water density. It is then possible, by inverting (3), to obtain the (average) surface around one particle track

(4) $$S=\frac{1}{\Phi }=\frac{1.6\cdot 10^{-9}\cdot LET}{D\cdot \rho }$$

and the corresponding value of $r_{max}$. By definition, this parameter represents the representative average distance between the particle tracks and it is, as a consequence, dose-rate-dependent. Returning to the initial-boundary value problem, it acquires the final form

(5) $$\left\{\begin{array}{c} \partial_{t}\left[A\right]\left(t,r\right)-D\partial_{r}^{2}\left[A\right]\left(t,r\right)-\frac{D}{r}\partial_{r}\left[A\right]\left(t,r\right)=0\\ \left[A\right]\left(t=0,r\right)=\left[A_{0}\right]\left(r\right)\;\mathrm{Initial}\mathrm{condition}\\ \partial_{r}\left[A\right]\left(t,r\right){{|}_{r=0}=\partial_{r}\left[A\right]\left(t,r\right)|}_{r=r_{max}}=0\;\mathrm{Boundary}\mathrm{conditions} \end{array}\right.$$

A finite difference approximation of the derivatives in a Crank–Nicolson scheme is applied. This is a numerical, implicit method used to compute values of approximate solutions of partial differential equations, where both the current ($\left[A\right]\left(t_{n},r\right)$) and consecutive ($\left[A\right]\left(t_{n+1},r\right)$) states of the system are used. Explicitly, considering a time step dt, discrete times $t_{n+1}=t_{n}+dt$ and using the notation $\left[A_{n}\right]\left(r\right)=\left[A\right]\left(t_{n},r\right)$, the differential Equation (5) becomes

(6) $$\frac{\left[A_{n+1}\right]\left(r\right)-\left[A_{n}\right]\left(r\right)}{dt}=\frac{1}{2}D\partial_{r}^{2}\left[A_{n+1}\right]\left(r\right)+\frac{1}{2}\frac{D}{r}\partial_{r}\left[A_{n+1}\right]\left(r\right)+\frac{1}{2}D\partial_{r}^{2}\left[A_{n}\right]\left(r\right)+\frac{1}{2}\frac{D}{r}\partial_{r}\left[A_{n}\right]\left(r\right)$$

endowed with the same initial and boundary conditions as (5). By approximating the radial derivatives in a finite-difference scheme, using the centres of the bins as a partition of the spatial domain $\left[0,r_{max}\right]$, it is possible to express Equation (6) by collecting the terms depending on $\left[A_{n+1}\right]\left(r\right)$ on the left side of the equation, whereas those depending on $\left[A_{n}\right]\left(r\right)$ are moved to the right side. Two “virtual” radial points are additionally introduced in order to ensure the validity of the partial differential equation at the boundary and to implement the boundary conditions. These are mathematical tools without physical relevance, and are used to implement the numerical method. By defining ${\vec{a}}_{n+1}$ and ${\vec{a}}_{n}$ as two vectors whose components are the concentration functions $\left[A_{n+1}\right]\left(r\right)$ and $\left[A_{n}\right]\left(r\right)$ evaluated at the bin centres, and due to the linearity of Equation (6), it is possible to recast the rewritten equation in a matrix (*M*, *N*) representation, in order to obtain the values of the distribution $\left[A_{n+1}\right]\left(r\right)$ at the bin centres after the time step dt

(7) $$M{\vec{a}}_{n+1}=N{\vec{a}}_{n}\to {\vec{a}}_{n+1}=M^{-1}N{\vec{a}}_{n}$$

with matrices *N* and *M* depending on the derivative approximations of (6). At this point, due to its tridiagonal form (i.e., with non-zero elements only in the main diagonal and in the first diagonals above and below it), *M* can be inverted with the algorithm proposed by [50], which exploits the principal minors of the matrix in a recursive way. The resulting product $M^{-1}N$ has been validated with MATLAB’s internal routine for matrix inversion, as shown in Figure S3 in the Supplementary Materials. Concerning the definition of the time step (dt) and the bin widths (bw, corresponding then to the distances between the bin centres) for the distributions, homogeneous time and space partitions are opted for, with dt = 0.5 ns and bw ≈ 20 nm, respectively.

As for the reaction’s resolution, an explicit algorithm (forward Euler method) is applied, by approximating the derivatives through a Taylor expansion. Given a simple reaction for species *A*, *B*, *C*

(8) A+B→C

with rate

(9) $$\frac{d[A](t,r)}{dt}=-\kappa_{AB}\left[A\right]\left(t,r\right)\left[B\right]\left(t,r\right)$$

the increment $\Delta A_{n}\left(r\right)$ for the species *A* at time $t_{n}$ can be defined as

(10) $$\Delta A_{n}\left(r\right)=-\kappa_{AB}\left[A_{n}\right]\left(r\right)\left[B_{n}\right]\left(r\right)dt$$

to obtain the distribution after the time step dt

(11) $$\left[A_{n+1}\right]\left(r\right)=\left[A_{n}\right]\left(r\right)+\Delta A_{n}\left(r\right)$$

by simply evaluating (11) at the bin centres. Firstly, the increments are defined, accounting for all the reactions both consuming and producing the species. These are specific for each locally homogeneous bin concentration (note the dependence on *r*). Subsequently, the distributions are updated. In summary, whereas the reactions are computed between the same bins, the diffusion process is calculated across the bins of one single species, in a similar fashion as [44].

To further improve the computational speed, especially at longer times, a control on the flattening of the radial concentrations has been introduced. Once the relative standard deviation featuring one distribution becomes lower than a user-specified threshold (e.g., 5%), the entire concentration is recast into one constant (flat) value. The diffusion step will then become unnecessary, and the overall calculation will be sped up (“phase III”).

### 4.2. Simulation Settings

Some additional comments have to be made regarding the simulation settings. Firstly, the sample thickness must be sufficiently small to fulfil the “track segment condition” (i.e., no significant LET variations inside the computed geometry for the primary radiation). Regarding the target dimensions, a cylindrical geometry with a radius of 5 µm has been chosen, meaning that any secondary particles exiting the target volume would not be tracked anymore. On the other hand, a target’s height of ∼10 µm is used for simulations with electron and proton beams, whereas smaller values of (1–2) µm are preferred for higher LET particles. At the same time, to prevent edge effects (enabling electron build up) [20], thin water phantoms (cylinders with 5 µm radius, 0.5 µm height) in front and behind the main one are introduced.

For oxygenated environments, the presence of the solute is processed as in the previous implementation [21]. The molecular oxygen is therefore treated as a continuum, i.e., as one uniform concentration value, regardless of the distance from the track centre. Additionally, variations in the global $O_{2}$ level within the target are not accounted for. This hypothesis is valid under low radiation-induced consumptions and may correspond, in a biological tissue, to a situation wherein blood oxygen resupply is faster than its removal by radicals. This requirement has been verified under conventional dose rates [51]. Concomitantly, its concentration must not be too high, in order to ignore direct interactions with radiation [20,33]. This value is computed by exploiting Henry’s law. In addition, the Noyes’ temporal term present in the coefficients for the two reactions with $O_{2}$ (second term in the parenthesis of Equation (10) in [21]) is removed from TRAX-CHEM, to be more consistent with TRAX-CHEMxt wherein the $O_{2}$ concentration is used explicitly rather than within a probability function, and the reactions are computed with a new algorithm (cf. end of Section 4.1). It can be noticed that oxygen in the environment starts to play a role within the reaction scheme at ∼1 ns [20,21], when the additional contribution coming from the Noyes’ term can be already neglected. This implies that the reaction rate can be approximated with a constant value.

The reactions are supposed to be in a diffusion-controlled regime, with rates determined under normal conditions, i.e., neutral pH and 25 °C. In TRAX-CHEMxt, both the rate constants and the diffusion coefficients are taken in water and are the same ones used in TRAX-CHEM [21] (Table S1 in the Supplementary Materials). Moreover, the pH is fixed at 7 and its effects on the acid-base pairs are not explicitly simulated. This is because non-neutral pH conditions and pH variations within the computed time are not yet implemented in the TRAX-CHEM code. In order to reduce the statistical uncertainties and fluctuations of the species’ concentration distributions, parallel calculations are performed. Depending on the LET, several hundred to several thousand (300–5000) independent tracks have been simulated, each impinging at the centre of the cylinder and parallel to its main axis. The result was then averaged to obtain the general behaviour for that specific beam quality. Fewer runs were needed for higher LET radiations as compared to low LET due to the increased amount of radicals produced.

### 4.3. Time Step and Transitional Time Point between TRAX-CHEM and TRAX-CHEMxt

The initial time step for TRAX-CHEMxt is chosen to be approximately equal to 0.5 ns. This dt was originally determined from a G-value analysis, by monitoring a 5% maximal change in the $OH^{•}$ curve (one of the most affected species by fluctuations, due to its high reactivity). However, a built-in function has been implemented to increase the time step automatically after some specific “flags”, with a maximum value of 10 ns for times longer than 1 ms. The list of all the time steps adopted is shown in Table 4. Every value was empirically proven to provide reliable results, by monitoring that the predictions match closely, both in terms of species distributions and G-values from TRAX-CHEM, at 1 µs and 10 µs. Fixing an upper limit on the time step avoids error amplifications due to the finite difference scheme applied, while also keeping spurious oscillations under control. A similar empirical analysis was carried out to select reliable bin widths, with a standard one of ≈20 nm.

The choice of the time point at which the information has to be handed over from one algorithm to the other is not trivial. This is due to the intrinsic differences in energy deposition of the various beam qualities simulated. Depending on the initial LET, the time at which the local homogeneity is reached within the histogram bins fluctuates significantly [44]. Transitions at early stages of the chemical evolution will result in unphysical outcomes (cf. deviations in Figure 2 for $t_{in}$ = 100 ns). On the other hand, a change between the Monte Carlo method and the concentration-based approach at advanced stages will make the entire simulation less efficient, due to the increasing computational costs, especially for high LET irradiations [44]. The optimal time point for a smooth transition between TRAX-CHEM and TRAX-CHEMxt has been found to lie around 500 ns (Section 2.1). Differences in the total number of species produced at 1 µs do not exceed 3–6%, as summarised in Table S2 in the Supplementary Materials. Moreover, the predicted spatial distributions and G-values agree closely (Section 2.4.1).

## 5. Conclusions

The new extension of the TRAX-CHEM code, TRAX-CHEMxt, is consistent with the current validated simulations, with on top the capacity of pushing the computation to longer times, maintaining trustable results, but faster. Moreover, it demonstrated the importance of keeping track of structure-based information also beyond the µs, to reliably simulate the chemical yields of intermediate and high LET radiations. The next step will be to simulate a more realistic cell-like environment and to benchmark the code predictions with additional experimental outcomes. Data on $H_{2}O_{2}$ production [43] or on $O_{2}$ depletion [51,52] may be good candidates in this regard. At the same time, validations with other simulations, such as those regarding the scavenging effect of GSH [33] or the production of organic peroxyl radicals under several irradiation conditions [32] will be carried out as well. A detailed study on the reaction network featuring different biomolecules may be insightful to understand the preferred reaction channels and competing pathways by which some compounds behave as protectors [5], and at the same time how other species act as radiation sensitisers [4]. Introducing these solutes may also be beneficial for the comprehension of the radiolysis-induced effects, and how these contribute to more complex, dose-rate-dependent mechanisms such as FLASH [15,42].

## Figures and Tables

**Table 1:** Yield predictions (molecules/100 eV) of reactants in anoxic conditions, ($H^{•}+e_{\mathrm{aq}}^{-}$), and the respective products in an oxic environment, ($HO_{2}^{•}+O_{2}^{•-}$), for a 500 keV electron beam and a 90 MeV proton beam at 1 µs after irradiation. The registered deviations are, respectively, 0.6% and 1%. In addition, the outcomes for a 90 MeV/u carbon beam are also shown. Initial data taken at $t_{in}$ = 500 ns.

Particle Type	pO${}_{2}$ (atm)	Species	Yield
500 keV e^−^	0%	$H^{•}+e_{\mathrm{aq}}^{-}$	3.19
	21%	$HO_{2}^{•}+O_{2}^{•-}$	3.21
90 MeV H	0%	$H^{•}+e_{\mathrm{aq}}^{-}$	3.12
	21%	$HO_{2}^{•}+O_{2}^{•-}$	3.15
90 MeV/u 12C	0%	$H^{•}+e_{\mathrm{aq}}^{-}$	1.40
	21%	$HO_{2}^{•}+O_{2}^{•-}$	1.56

**Table 2:** Computational time (τ) required by TRAX-CHEM and TRAX-CHEMxt to obtain the data at 10 µs, from different beam qualities and oxygenation conditions. Furthermore, an additional τ for the novel extension at 1 ms, and a computational gain at 10 µs for each scenario are also displayed.

Initial Conditions	τ(10 µs)—TRAX-CHEM ^1^	τ(10 µs)—TRAX-CHEMxt	τ(1 ms)—TRAX-CHEMxt	Computational Gain (10 µs)
500 keV e^−^, 1% pO${}_{2}$	∼12 h	75 s	27 min	1369
150 MeV/u 12C, 7% pO${}_{2}$	>2 days	80 s	30 min	2189

^1^ CPU time, averaged over parallel calculations, to compute 500 and 20 events for, respectively, 500 keV electrons and 150 MeV/u carbon ions. Multithreading was not used, whereas instead trivial parallelisations were performed on a cluster featured by multiple machines with processors from IBM Power8 and above. The RAM exploited was between 2 GB and 8 GB.

**Table 3:** Chemical yields (molecules/100 eV) determined at 10 µs in a 21% oxygenated water environment. “Experiment”: experimental data from [39]; “Colliaux et al.”: simulation with γ-Co${}^{60}$ from [20]; “This work”: values from TRAX-CHEMxt, with 500 keV electrons and initial radial distributions taken at 600 ns.

Species	Experiment [39]	Colliaux et al. [20]	This Work
$OH^{•}$	2.7	2.8	2.7
$HO_{2}^{•}$ + $O_{2}^{•-}$	3.3	3.4	3.2
$H_{2}O_{2}$	0.67	0.61	0.61

**Table 4:** List of all the time steps, dt, assigned to the various time intervals.

Time Interval	dt (ns)
$t_{in}$–1 µs	0.5
1 µs–10 µs	1
10 µs–100 µs	2.5
100 µs–1 ms	5
1 ms–$t_{end}$	10

## Data Availability

All the relevant data are contained within the article and its Supporting Information files. Further data on the code and on its advanced features, together with the possibility to download a stable version of TRAX-CHEM, are available upon request from the corresponding author.

## Supplementary Materials

The supporting information can be downloaded at https://www.mdpi.com/article/10.3390/ijms24119398/s1.

## Abbreviations

The following abbreviations are used in this manuscript:

e-	Electron beam
H	Proton beam
12C	Carbon ion beam
LET	Linear energy transfer
IRT	Independent reaction time
B.C.	Boundary conditions
SOD	Superoxide dismutase
CAT	Catalase
GSH	Glutathione
