# Supplementary material for: TRAX-CHEMxt: Towards the Homogeneous Chemical Stage of Radiation Damage
Source: Int J Mol Sci. 2023 May 28;24(11):9398. doi: 10.3390/ijms24119398 (PMC10253526; doi:10.3390/ijms24119398)
Supplement: Supplementary file 1 [file ijms-24-09398-s001.zip › ijms-2343965-supplementary.pdf]

Supplementary Materials: TRAX-CHEMxt: Towards the Homogeneous Chemical Stage of Radiation Damage

Gianmarco Camazzola, Daria Boscolo, Emanuele Scifoni, Alexander Dorn, Marco Durante, Michael Krämer, Valentino Abram and Martina C. Fuss

Table S1. List of reactions, respective reaction rate constants and diffusion coefficients implemented in TRAX-CHEMxt, identical to the ones simulated in TRAX-CHEM. The κ values are determined under normal conditions, i.e. neutral pH and 25 °C. Adapted from [1].

	**Reaction**	κ **(10^{10}dm^{3}mol^{-1}s^{-1})**
(i)	$OH^{\bullet} + OH^{\bullet} \rightarrow H_2O_2$	0.6
(ii)	$OH^{\bullet} + e^-_{aq} \rightarrow OH^-$	2.2
(iii)	$OH^{\bullet} + H^{\bullet} \rightarrow H_2O$	2.0
(iv)	$OH^{\bullet} + H_2 \rightarrow H^{\bullet} + H_2O$	0.0045
(v)	$OH^{\bullet} + H_2O_2 \rightarrow HO_2^{\bullet} + H_2O$	0.0023
(vi)	$e^-_{aq} + e^-_{aq} + H_2O + H_2O \rightarrow H_2 + OH^- + OH^-$	0.55
(vii)	$e^-_{aq} + H^{\bullet} + H_2O \rightarrow H_2 + OH^-$	2.5
(viii)	$e^-_{aq} + H_3O^+ \rightarrow H^{\bullet} + H_2O$	1.7
(ix)	$e^-_{aq} + H_2O_2 \rightarrow OH^{\bullet} + OH^-$	1.0
(x)	$H^{\bullet} + H^{\bullet} \rightarrow H_2$	1.0
(xi)	$H^{\bullet} + H_2O_2 \rightarrow OH^{\bullet} + H_2O$	0.01
(xii)	$H^{\bullet} + OH^- \rightarrow e^-_{aq} + H_2O$	0.002
(xiii)	$H_3O^+ + OH^- \rightarrow H_2O + H_2O$	10.0
(xiv)	$e^-_{aq} + O_2 \rightarrow O_2^{\bullet -}$	1.9
(xv)	$H^{\bullet} + O_2 \rightarrow HO_2^{\bullet}$	2.0
(xvi)	$OH^{\bullet} + HO_2^{\bullet} \rightarrow O_2 + H_2O$	1.0
(xvii)	$OH^{\bullet} + O_2^{\bullet -} \rightarrow O_2 + OH^-$	0.9
(xviii)	$OH^{\bullet} + HO_2^- \rightarrow HO_2^{\bullet} + OH^-$	0.5
(xix)	$e^-_{aq} + HO_2^{\bullet} \rightarrow HO_2^-$	2.0
(xx)	$e^-_{aq} + O_2^{\bullet -} + H_2O \rightarrow OH^- + HO_2^-$	1.3
(xxi)	$H^{\bullet} + HO_2^{\bullet} \rightarrow H_2O_2$	2.0
(xxii)	$H^{\bullet} + O_2^{\bullet -} \rightarrow HO_2^-$	2.0
(xxiii)	$H_3O^+ + O_2^{\bullet -} \rightarrow HO_2^{\bullet} + H_2O$	3.0
(xxiv)	$H_3O^+ + HO_2^- \rightarrow H_2O_2 + H_2O$	2.0
(xxv)	$HO_2^{\bullet} + HO_2^{\bullet} \rightarrow H_2O_2 + O_2$	0.000076
(xxvi)	$HO_2^{\bullet} + O_2^{\bullet -} \rightarrow O_2 + HO_2^-$	0.0085

**Species**	**D (10^{-9}m^{2}s^{-1})**
$OH^{\bullet}$	2.8
H_3O^+	9.0
$H^{\bullet}$	7.0
e^-_{aq}	4.5
H_2	4.8
H_2O_2	2.3
OH^-	5.0
O_2	2.1
$HO_2^{\bullet}$	2.0
$O_2^{\bullet -}$	2.1
HO_2^-	2.0

Table S2. Deviations associated with TRAX-CHEMxt, for all the initial conditions simulated so far. Each deviation is derived by taking the biggest value from the differences between the total number of every radical and molecule predicted by the extension (with initial concentrations taken at 600 ns) and the respective quantities produced by TRAX-CHEM, divided by the latter. To determine these values, the contributions from HO_2^- have been disregarded due to their very low yields.

Particle type	Energy	pO_2 (atm)	Deviation at 1 µs
Electrons	500 keV	0%	1%
Electrons	500 keV	0.5%	1%
Electrons	500 keV	1%	1%
Electrons	500 keV	3%	1%
Electrons	500 keV	7%	1%
Electrons	500 keV	21%	2%
Electrons	1 MeV	0%	1%
Protons	40 MeV	0%	1%
Protons	65 MeV	21%	2%
Protons	90 MeV	0%	1%
Protons	90 MeV	4%	1%
Protons	90 MeV	21%	2%
Helium ions	150 MeV/u	21%	2%
Carbon ions	10 MeV/u	21%	6%
Carbon ions	20 MeV/u	21%	6%
Carbon ions	40 MeV/u	0%	4%
Carbon ions	40 MeV/u	3%	5%
Carbon ions	40 MeV/u	21%	5%
Carbon ions	90 MeV/u	0%	4%
Carbon ions	90 MeV/u	0.5%	4%
Carbon ions	90 MeV/u	2%	4%
Carbon ions	90 MeV/u	3%	4%
Carbon ions	90 MeV/u	7%	5%
Carbon ions	90 MeV/u	21%	5%
Carbon ions	150 MeV/u	7%	4%
Carbon ions	300 MeV/u	21%	3%

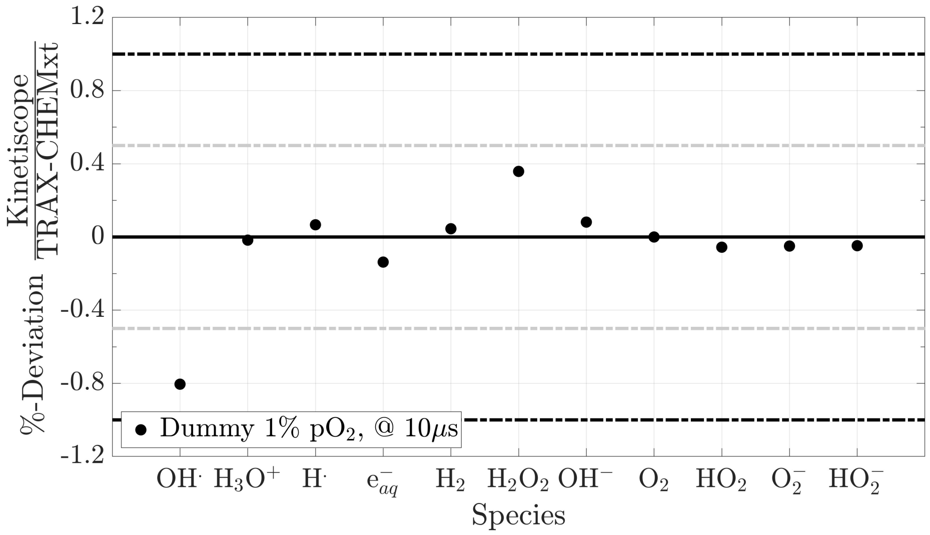

Figure S1. Deviations between Kinetiscope and TRAX-CHEMxt, registered for a "dummy" case with uniform concentrations assigned randomly to each chemical species, in a water environment with pO_2 = 1%. Marked with dash-dotted lines are discrepancies of ± 0.5% and ± 1%. Initial data handed over to both codes at 1 µs, and the chemical network in this reaction-limited domain was simulated up to 10 µs.

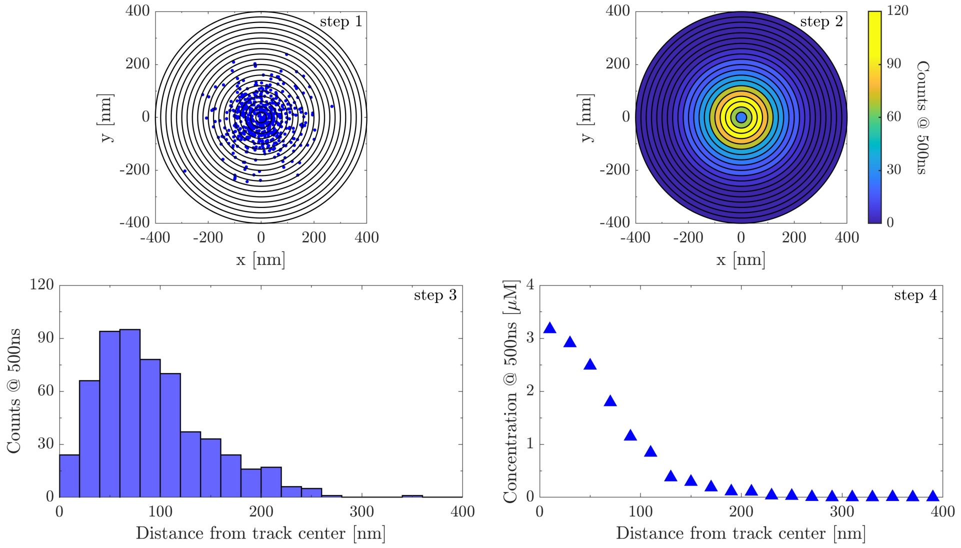

Figure S2. Steps constituting the conversion of the information from TRAX-CHEM to TRAX-CHEMxt. After collecting the positions of a specific radical or molecule around the track center (step 1) and counting its amount within each radial bin (step 2), the respective histogram is created (step 3). In conclusion, the various counts are converted into concentration values (step 4), supposing thus a uniform concentration within each radial bin. In the proposed example, a bin width and a transitional time of respectively 20 nm and 500 ns are used. The concentrations are assigned to the respective bin centers.

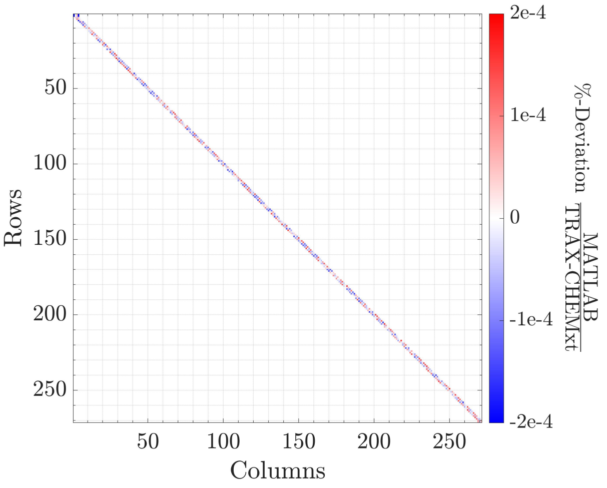

Figure S3. Deviations between the matrix values for the product $M^{-1}N$, computed by MATLAB and the implemented algorithm in TRAX-CHEMxt following [2], for the diffusion of the hydroxyl radical. The set of parameters exploited is: time step $dt = 5{\cdot}10^{-10}$ s, diffusion coefficient $D = 2.8{\cdot}10^{9}$ nm^2/s, bin width (distance between two consecutive radii) $bw \approx 20$ nm. The error is on the order of 10^{-6}.

References

1. Boscolo, D.; Krämer, M.; Fuss, M.C.; Durante, M.; Scifoni, E. Impact of target oxygenation on the chemical track evolution of ion and electron radiation. *Int. J. Mol. Sci.* **2020**, *21*, 424. https://doi.org/10.3390/ijms21020424.
2. Usmani, R.A. Inversion of Jacobi's tridiagonal matrix. *Comput. Math. Appl.* **1994**, *27*, 59–66. https://doi.org/10.1016/0898-1221(94)90066-3.

Disclaimer/Publisher's Note: The statements, opinions and data contained in all publications are solely those of the individual author(s) and contributor(s) and not of MDPI and/or the editor(s). MDPI and/or the editor(s) disclaim responsibility for any injury to people or property resulting from any ideas, methods, instructions or products referred to in the content.
